# A Literature Mini-Review of Transcranial Direct Current Stimulation in Schizophrenia

**DOI:** 10.3389/fpsyt.2022.874128

**Published:** 2022-04-21

**Authors:** Zuzana Stuchlíková, Monika Klírová

**Affiliations:** ^1^National Institute of Mental Health, Klecany, Czechia; ^2^Third Faculty of Medicine, Charles University, Prague, Czechia; ^3^Hospital České Budĕjovice, a.s., České Budĕjovice, Czechia

**Keywords:** review, neurostimulation, direct current stimulation, tDCS, schizophrenia, schizophrenic

## Abstract

Transcranial direct current stimulation (tDCS) is a non-invasive neurostimulation method that utilizes the effect of low-current on brain tissue. In recent years, the effect of transcranial direct current stimulation has been investigated as a therapeutic modality in various neuropsychiatric indications, one of them being schizophrenia. This article aims to provide an overview of the potential application and effect of tDCS in treating patients with schizophrenia. A literature search was performed using the PubMed, Web of Science, and Google Scholar databases for relevant research published from any date until December 2021. Eligible studies included those that used randomized controlled parallel-group design and focused on the use of transcranial direct current stimulation for the treatment of positive, negative, or cognitive symptoms of schizophrenia. Studies were divided into groups based on the focus of research and an overview is provided in separate sections and tables in the article. The original database search yielded 705 results out of which 27 randomized controlled trials met the eligibility criteria and were selected and used for the purpose of this article. In a review of the selected trials, transcranial direct current stimulation is a safe and well-tolerated method that appears to have the potential as an effective modality for the treatment of positive and negative schizophrenic symptoms and offers promising results in influencing cognition. However, ongoing research is needed to confirm these conclusions and to further specify distinct application parameters.

## Introduction

Schizophrenia is a serious mental illness with an average lifetime prevalence of 6.35 per 1,000 persons (1). Symptoms of schizophrenia may be divided into separate clusters in three main domains that are represented by positive, negative, and cognitive symptoms. Whereas the pharmacological approach is effective mainly in the treatment of positive symptoms, it shows only small benefits in treating negative and cognitive symptoms (2). This is one of the reasons why the scientific focus remains on researching and improving new treatment options and therapeutic modalities. Attenuated cortical activity in prefrontal regions, i.e., hypofrontality (3, 4), and altered inter and intrahemispheric connectivity were described in individuals diagnosed with schizophrenia (5). Frontotemporal and frontoparietal disconnectivity is associated with negative (3, 6) and positive symptoms (3, 7). Hypoactivity of the prefrontal cortex and the disruption of its connection with temporoparietal and contralateral regions were described in relation to the cognitive symptoms (8–10). One of the treatment methods researched for schizophrenia is transcranial direct current stimulation (tDCS) due to its possible effects on the described disrupted cortical mechanisms.

Transcranial direct current stimulation is a non-invasive neuromodulation method based on the use of low-intensity direct current (usually 1–2 mA) and its effect on brain tissue (11). The direct current generated between the surface of electrodes (anode and cathode) placed on the scalp creates cortical changes dependent on the polarity of the applied current. During anodal tDCS (located under the anode), the depolarization of neuronal membranes occurs and thus the cortical excitability rises, meanwhile cathodal tDCS (under the cathode) has the opposite effect (12). Albeit the precise mechanism of the post-modulatory effect of tDCS remains not fully clarified, studies show that direct current stimulation may influence synaptic plasticity and affect remote brain regions by acting on non-synaptic axonal levels (13). The post-modulatory effect on the synaptic level is mediated through the alteration of Ca^2+^- dependent channels of N-methyl-D-aspartate (NMDA) and amino-3-hydroxy-5-methyl-4-isoxazolepropionate (AMPA) receptors and also through modulating GABA and its interaction with the glutamatergic system (12). These processes further influence brain-derived neutotrophic factor (BDNF) production leading to long-term potentiation (anodal tDCS) or depression (cathodal tDCS) (14, 15), and produce post-modulatory synaptic changes, with long-term potentiation strengthening connections between neurons and long-term depression weakening them (16). Recent research shows BDNF polymorphism to have an impact on the subject sensitivity to tDCS effects (17, 18). Other studies demonstrate a non-synaptic mechanism of tDCS after-effects based on changes in neuronal membrane function (19). tDCS also shows the ability to interfere with functional connectivity, synchronization, and oscillatory action of different cortical and subcortical neuronal networks (16, 17). Additional line of research focuses on possible immunomodulatory effects of tDCS and their importance in overall outcomes (20, 21). Furthermore, tDCS is considered to is considered to also act through induced epigenetic changes, such as histone acetylation and methylation (22, 23).

In recent years, tDCS has been explored as a possible treatment modality for a number of neurological and neuropsychiatric disorders (16, 24). Some of the areas of focus for tDCS application include neurodegenerative diseases such as Parkinson’s disease (25), motor rehabilitation (26), or cognitive improvement (27). One of the investigated disorders in connection to tDCS is also schizophrenia. Previous reviews mainly focused on tDCS efficacy in separate schizophrenic symptom groups such as auditory hallucinations (AH) (28), negative and cognitive symptoms (29, 30). This literature review focuses specifically on the therapeutic application of tDCS in patients with schizophrenia and aims to provide a comprehensive review of tDCS application and its effect on all schizophrenic symptom clusters. Contrary to recent guidelines (24), studies focusing on cognitive function in patients with schizophrenia were also included in this review.

## Materials and Methods

For the purpose of this review, a systematic literature search was performed using electronic databases, namely PubMed and Web of Science, and the Google Scholar search engine. The database search was performed on August 4th and on December 14th, 2021. The second search was performed to identify as recently published trials as possible, providing one additionally selected study. With the use of Boolean operators *(tdcs OR “transcranial direct current stimulation” OR “direct current stimulation”) AND (schizophrenia OR “schizophrenic disorder” OR schizophrenic)* the search yielded 318 results in the Pubmed database and 381 results in the Web of Science database. The filter to exclude meeting abstracts was used in the search. Subsequently, the Google Scholar search engine was used to identify six more sources from the last year not yet available in the databases.

Inclusion criteria were determined based on the population, intervention, control group, study design, and language used for publication. The population included adults diagnosed with schizophrenia or schizoaffective disorder. The intervention was defined as the use of tDCS for the treatment of positive, negative, or cognitive symptoms of schizophrenia. The control group was set as a sham tDCS application. The study design included randomized blinded studies with parallel arms, therefore excluding any open-label or cross-over trials. Only trials published in English were included. A total of 705 search results were screened based on the titles and/or abstracts. If a trial met inclusion criteria, the full text of the article was retrieved. Full texts were read and reviewed by the first investigator (ZS) with supervision by the senior investigator (MK) who provided subsequent clarification if necessary. Twenty-seven clinical trials were selected following the previously described procedure. A PRISMA flow diagram is available in the Supplementary Material.

Selected trials underwent qualitative analysis. Trials were divided into three groups based on the primary focus on positive, negative, or cognitive symptoms. Studies were categorized into the positive symptom group if the outcomes focused on changes in AH or PANSS total and positive scores. The negative symptom group included outcomes measured by SANS or PANSS negative score. Trials in the cognitive symptom group focused on changes in at least one observed cognitive outcome. Specific stimulation parameters such as total number and frequency of tDCS applications, electrode positioning, and intensity of the electrical current were assessed for each trial. The number of participants in both active and sham stimulation was identified. And primary and secondary clinical outcomes were highlighted. All the information was recorded in comprehensive tables.

The risk of bias was assessed for each of the included trials using the revised Cochrane risk of bias assessment tool (31).

## Results

Selected studies focused on the possible effect of tDCS application on the frontotemporal and bifrontal disconnectivity in schizophrenia and on ameliorating schizophrenic symptoms. Different positioning of the electrodes and stimulation protocols were explored. Most of the randomized controlled trials (RCTs) focused on the clinical effect of the stimulation, some of the research also focused on functional changes in distinct brain areas.

### Positive Symptoms

Treatment in schizophrenia is often focused on reducing AH as they are frequently present in patients and are often refractory to antipsychotic drugs. Eight of the identified RCTs focused on the reduction of AH as the primary outcome. Two additional trials investigated the effect of tDCS on the reduction of AH in relation to possible modulation of the disrupted neuronal processes in schizophrenia. All 10 studies used similar electrode placement with the anode positioned over the left prefrontal cortex and the cathode over the temporoparietal area. In addition, one study used a bilateral form of stimulation with the second pair of electrodes placed over corresponding contralateral positions (32). All of the studies used the current intensity of 2 mA.

Most of the studies applied tDCS twice daily on five consecutive days (33–39). Two protocols chose to apply stimulation only once per day (32, 40), and two protocols continued to administer stimulation for several weeks (32, 41).

The selected RCT studies do not provide consistent results regarding the efficacy of tDCS on AH intensity. A 2012 study (33) was one of the first to show a significant effect of tDCS in this indication. The effect on AH was also documented in five other studies (34–36, 39, 41). As a secondary outcome, the stimulation protocols reduced the Positive and Negative Syndrome Scale (PANSS) scores (33), and an improvement in working memory was described (41).

Four of the selected studies failed to demonstrate a significant effect of active tDCS stimulation on AH, despite a sufficient sample size and statistical significance being reached (32, 37, 38, 40). Furthermore, two of these RCT studies failed to confirm a significant effect in favor of active stimulation over placebo on any other of the observed symptoms (38, 40). The largest negative study in influencing AH, however, documents improvement in the observed PANSS score and in the level of insight (37). Data from this study were additionally analyzed in two subsequent publications (42, 43). The first paper commented on a trend-level improvement in planning ability, and further specified the positive trends in PANSS score change, where the amelioration of total and general psychopathology did not reach statistical significance compared to sham stimulation (42). The publication in the following year focused on the observed temporary improvement in insight, treatment adherence, and psychological domain of quality of life (43).

Increased activity in the frontal and temporoparietal cortex was previously described in relation to AH (44). A 2016 study (35) showed the effect of tDCS on the resting-state functional connectivity between the frontal and temporoparietal cortex. The reduction of aberrant connectivity positively correlated with the reduction of AH severity (35). These findings may help clarify the positive effect of the preferred frontotemporal stimulation used to improve AH. Subsequent analysis of brain activity in a selected sample from the previous study suggested that the strength of the tDCS-induced electric field reaching the left transverse temporal gyrus may have an important influence on the outcome of frontotemporal stimulation (45).

Impairment of the ability to distinguish between self-generated events and external stimuli was also described in relation to AH in patients with schizophrenia (46). One of the studies showed frontotemporal stimulation to be effective in improving the source-monitoring ability and the improvement positively correlated with a reduction in AH severity (34). A study published in 2019 (47) also explored tDCS application and its ability to influence source-monitoring deficits in a sample of subjects from the 2018 study (36), the findings documented improvement in corollary discharge.

An overview of the selected RCT studies, stimulation parameters, and observed effects is provided in Table 1.

**TABLE 1:** Effects of tDCS on positive symptoms.

Studies	Design	Inclusion criteria, diagnosis	Number of subjects (*n*)	Electrode placement Size of electrodes	Stimulation parameters *(duration, course, total number, intensity used)*	Sham parameters	Outcomes
Brunelin et al. 2012 (33)	RDBS, SH	SZ + TR AH	*n* = 30 (15 active, 15 sham)	Anode – F3/FP1 cathode – T3/P3 size: 7 × 5 cm	20 min 2x/day, 5 days (10) 2 mA	2 mA for 40 s +110 μA pulse over 15 ms every 550 ms	Robust AH reduction (mean improvement of 31% in AHRS score) lasting up to 3 months. Amelioration of schizophrenia symptoms as assessed by total PANSS, with significant effect on the negative dimension. Medium effect size on the positive and depressive dimension short of statistical significance.
Fitzgerald et al. 2014 (32)	2x RDBS, SH	SZ/SZA + persistent AH and negative symptoms	*n* = 24 (11 bilateral, 13 unilateral)	Unilateral: anode – F3 cathode – TP3 Bilateral: + anode – F4 + cathode – TP4 size: 35 cm^2^	20 min 1x/day, 3 weeks, weekdays (15) 2 mA	2 mA for 30 s with ramp-up	No substantial change in AH, PANSS or SANS score after neither unilateral nor bilateral stimulation.
Mondino et al. 2015 (34)*^a^*	RDBS, SH	SZ + TR AH	*n* = 28 (15 active, 13 sham)	Anode – F3/FP1 cathode- T3/P3 size: 7 × 5 cm	20 min 2x/day, 5 days (10) 2 mA	2 mA for 30 s	Medium effect on covert/overt speech misattributions in the active group. A large effect on AH frequency in the active group. The reduction in covert/overt speech misattributions positively correlated with the reduction in AH frequency.
Fröhlich et al. 2016 (40)	RDBS, SH	SZ/SZA + AH	n = 26 (13 active, 13 sham)	anode - F3/FP1 cathode - T3/P3 (+ return electrode Cz). size: 7 × 5cm	20 min 1x/day, 5 days (5) 2 mA	2 mA for 40 s	Lack of efficacy of active tDCS. A significant reduction in AH not specific to the treatment group. No significant change in PANSS.
Mondino et al. 2016 (35)*^b^*	RDBS, SH	SZ + TR AH	*n* = 23 (11 active, 12 sham)	Anode – F3/FP1 cathode – T3/P3 size: 7 × 5 cm	20 min 2x/day, 5 days (10) 2 mA	2 mA for 30 s	Significant reduction of AH as well as negative symptoms after active tDCS. Reduced resting state functional connectivity (rs-FC) of the left temporoparietal junction with the left anterior insula and the right inferior gyrus, and increased rs-FC of the left TPJ with the left angular gyrus, the left DLPFC and the precuneus after active tDCS.
Bose et al. 2018 (36)	RDBS, SH + open label extension (OLE)	SZ + TR AH	*n* = 25 (12 active, 13 sham)	Anode – F3/FP1 cathode – T3/P3 size: 7 × 5 cm	20 min 2x/day, 5 days (10) 2 mA	2 mA for 40 s + 110 μA pulse over 15 ms every 550 ms	RDBS phase: Significant tDCS-type X time-point interaction with significantly greater reduction of AHRS score in active tDCS (30.22%). OLE phase: Significant greater reduction in AH severity in sham-to-verum crossed over patients.
Chang et al. 2018 (37)	RDBS, SH	SZ/SZA + TR AH	*n* = 60 (30 active, 30 sham)	Anode – F3/FP1 cathode – T3/P3 size: 7 × 5 cm	20 min 2x/day, 5 days (10) 2 mA	2 mA for 30 s	No significant changes in the severity of AH or in PANSS after active tDCS. Improvement in the level of insight into illness and into positive symptoms lasting 1 month after active tDCS.
Koops et al. 2018 (38)	RDBS, SH	TR AH (several diagnostic categories)	*n* = 54 (28 active, 26 sham)	Anode – FP1/F3 cathode – T3/P3 size: 7 × 5 cm	20 min 2x/day, 5 days (10) 2 mA	2 mA for 40 s +110 μA pulse over 15 ms every 550 ms	Active tDCS was not more effective than placebo on any of the main outcomes *(AHRS, PANSS, the Stroop, and the Trail Making Test*).
Kantrowitz et al. 2019 (39)	RDBS, SH	SZ/SZA + TR AH	*n* = 89 (47 active, 42 sham)	Anode – F3/FP1 cathode – T3/P3 size: 6.75 × 5.75 cm	20 min 2x/day, 5 days (10) 2 mA	2 mA for 40 s ramp-up/ramp-down	Significant reduction in AHRS total score (>30%) across 1-week and 1-month. (Greatest change observed on the AHRS loudness item.) No significant change in PANSS negative.
Lindenmayer et al. 2019 (41)	RDBS, SH	Ultra-TR SZ +persistent AH	*n* = 28 (15 active, 13 sham)	Anode – F3/FP1 cathode – T3/P3 size: 7 × 5 cm	20 min 2x/day, 4 weeks, weekdays (40) 2 mA	2 mA for 40 s +110 μA pulse over 15 ms every 550 ms	Small but meaningful AHRS reduction (21.9%). Significant change in working memory and PANSS total in the active tDCS group. No significant changes in PANSS subscales.


Gomes et al. 2015 (53)	RDBS, SH	SZ	*n* = 15 (7 active, 8 sham)	Anode – F3 cathode – F4 size: not specified	20 min 1x/day, 2 weeks, weekdays (10) 2 mA	Not specified	PANSS reduction (total score 42.3%, negative score 45.4%, general score 29%) after active tDCS. No effects for CDSS, GAF and PANSSpositive score.
Smith et al. 2015 (66)	RDBS, SH	SZ/SZA + current smokers	*n* = 37 (19 active, 18 sham)	Anode – F3 cathode – FP2 size: 5.08 cm^2^	20 min 1x/day, 5 days (5) 2 mA	2 mA for 40 s	Significant improvements in MCCB Composite score and the domain scores for Working Memory and Attention Vigilance with large effect sizes. (MCCB Composite score and domain score for Working Memory remained significant with corrected significance levels). No statistically significant effects on secondary outcome measures (PANSS scores, hallucinations, cigarette craving, or cigarettes smoked).
Palm et al. 2016 (54)	RDBS, SH	SZ with predominantly negative symptoms	*n* = 20 (10 active, 10 sham)	Anode – F3 cathode – FP2 size: 7 × 5 cm	20 min 1x/day, 2 weeks, weekdays (10) 2 mA	Not specified	Significantly greater decrease in SANS score (36.1%) and PANSS sum scores (23.4%) after active tDCS compared to sham (0.7%, 2.2% respectively). Explorative analysis of fcMRI data revealed changes in subgenual cortex and DLPFC connectivity within fontal-thalamic-temporo-parietal networks. No significant effect of active tDCS on CDSS score or cognitive outcomes.
Shiozawa et al. 2016 (70)	RDBS, SH	SZ	*n* = 10 (5 active, 5 sham)	anode – left DLPFC cathode – right DLPFC size: 35 cm^2^	20 min 2x/day, 5 days 1x “online” tDCS (10) 2 mA + *cognitive training randomly applied during one of the tDCS sessions*	2 mA for 60 s	Failed to demonstrate effect of “online tDCS” on improvement in clinical outcomes (N-back and sequence learning task, PANSS).
Gomes et al. 2018 (69)	RDBS, SH	SZ	*n* = 24 (12 active, 12 sham)	Anode– left DLPFC cathode – right DLPFC size: 25 cm^2^	20 min 1x/day, 2 weeks, weekdays (10) 2 mA	2 mA for 30 s	Without improvement in working memory. Therapeutic effects of tDCS for treatment of persistent symptoms in schizophrenia, with reduction of negative symptoms.
Jeon et al. 2018 (68)	RDBS, SH	SZ	*n* = 56 (28 active, 28 sham)	Anode – F3 cathode – F4 size: 25 cm^2^	30 min 1x/day, 2 weeks, weekdays (10) 2 mA	2 mA for 30 s ramp-up + 30 s ramp-down	MCCB working memory and overall scores improved over time after active tDCS. Depressive symptoms decreased after tDCS. Improvement of PANSS score (did not reach statistical significance).
Bose et al. 2019 (47)*^c^*	RDBS, SH *ancillary study*	SZ + TR AH	*n* = 13 (7 active, 6 sham)	Anode –left DLPFC cathode – left TPJ (no further specification)	20 min 2x/day, 5 days (10) 2 mA	2 mA for 40 s +110 μA pulse over 15 ms every 550 ms	Improvements in corollary discharge with concurrent reduction in AH scores after active tDCS. Change in corollary discharge correlated with change in AH severity.
Weickert et al. 2019 (74)	RDBS, SH	SZ/SZA	*n* = 12 (6 active, 6 sham)	Anode – F4 cathode – T3/P3 size: 7 × 5 cm	20 min 1x/day, 4 weeks, weekdays “online” tDCS (20) 2 mA + 2-back test	2 mA for 15 s ramp-up + 15 s ramp-down	Significant improvement in language-based working memory after 2 weeks and verbal fluency after 2 and 4 weeks. No significant effect on any other cognitive assessment. No significant effects on AHRS score.
Chang et al. 2020 (55)	RDBS, SH	SZ/SZA	*n* = 60 (30 active, 30 sham)	Bilateral: anode 1 – F3/FP1 anode 2 – F4/FP2 reference electrodes – ipsilateral forearm size: 7 × 5 cm	20 min 2x/day, 5 days (10) 2 mA	2 mA for 30 s +110 μA pulse over 15 ms every 550 ms	Rapid reduction of negative symptoms measured by PANSS, with the beneficial effect lasting up to 3 months. Improvement of psychosocial functioning. Improvement of psychopathological symptoms especially for disorganization and cognitive symptoms as measured by the PANSS. No effects on other schizophrenia symptom dimensions or on the performance in neurocognitive tests.
Smith et al. 2020 (65)	RSBS, SH	SZ + significant cognitive deficit	*n* = 49 (24 active, 25 sham) *45 evaluated	Anode – F3 cathode – FP2 size: 5,08 cm^2^	20 min 1x/day, 2 weeks, weekdays (10) 2 mA	2 mA for 40 s	Significant pro-cognitive effects on some aspects of cognitive testing at 2 and 4 weeks after the final tDCS session (MATRICS Speed of Processing domain). No immediate pro-cognitive effects. No significant effects on other psychiatric outcomes.
Valiengo et al. 2020 (52)	RDBS, SH	SZ	*n* = 100 (50 active, 50 sham)	Anode – F3 cathode – T3/P3 size: 7 × 5 cm	20 min 2x/day, 5 days (10) 2 mA	2 mA for 30 s + 40 s ramp-up + 40 s ramp-down	Significantly greater improvement in PANSS scores after active tDCS. Higher response rates for negative symptoms in the active group.
Dharani et al. 2021 (56)	RDBS, SH	SZ	*n* = 14 (7 active, 7 sham)	Anode – F3 4 return electrodes (FC1, F7, FC5, AF3) size: 1 cm radius ring electrodes	20 min 2x/day, 5 days (10) 2 mA	1 mA for 30 s	Significant reduction in PANSS, SANS, and CGI-S.
Meiron et al. 2021 (71)	RDBS, SH	SZ/SZA	*n* = 19 (11 active, 8 sham, +12 healthy controls for baseline and post-tDCS comparison)	Anode – F3/AF3 cathode – vertex size: 5 × 5 cm	20 min 2x/day, 5 days (10) 2 mA	2 mA for 30 s ramp-up + 30 s ramp-down	Improvement in working memory performance in the active tDCS group. Post-tDCS scores were comparable to healthy control scores. Significant alleviation of symptom severity maintained for four weeks.
Mondino et al. 2021 (45)*^d^*	RDBS, SH *Electrical field modeling using baseline structural MRI scans*	SZ + TR AH	*n* = 17 subjects with active tDCS (6 responders, 11 non-responders)	Anode – F3/FP1 cathode – T3/P3 size: 7 × 5 cm	20 min 2x/day, 5 days (10) 2 mA	2 mA for 30 s	Higher electric field strength in the left transverse temporal gyrus at baseline in responders to tDCS (at least a 50% decrease of AH 1 month after the last tDCS session) compared to non-responders.

*Studies placed below the double line did not explore positive symptoms as the primary outcome. AH, auditory hallucinations; AHRS, Auditory Hallucinations Rating Scale; DLPFC, dorsolateral prefrontal cortex; MCCB, MATRICS Consensus Cognitive Battery; OL, open label; PANSS, Positive and Negative Syndrome Scale; RDBS, randomized double blind study; RSBS, randomized single blind study; SH, sham controlled; SZ, schizophrenia; SZA, schizoaffective disorder; TPJ, temporoparietal junction; TR, treatment resistant. ^a^The sample partially overlaps (n = 15) with an already published study (34). ^b^Clinical data from 7 patients of the sham group and 8 of the active group were already used (35). ^c^A random subset of a previous clinical study (47). ^d^Data from 11 patients were used in a previously published study (45).*

### Negative Symptoms

Key negative symptoms of schizophrenia include blunted affect, alogia, anhedonia, asociality, and avolition (48). Negative symptoms may present as one of the first symptoms of schizophrenia (49) and most antipsychotic drugs have a limited effect on their treatment (50). The search yielded five RCT studies mainly focusing on tDCS application as a possible treatment for negative symptoms. All of the trials demonstrated active stimulation to be at least partially effective in improving the observed outcomes. Based on the assumed association between negative symptoms and neurobiological correlates in the prefrontal cortex (51), the anode was positioned over the corresponding area in all of the studies. One trial protocol placed the cathode over the ipsilateral temporoparietal cortex (52), two over the contralateral prefrontal cortex (53, 54), one protocol used bi-anodal stimulation of the prefrontal cortex bilaterally with cathodes placed on the forearms (55), and one trial used high definition tDCS (HD-tDCS) with four return electrodes positioned around the anode (56). This was the only study using HD-tDCS to be included in this review. All of the selected studies used current intensity of 2 mA for stimulation.

The study using bi-anodal stimulation presented positive outcomes in psychosocial functioning and ameliorated disorganization and cognitive symptoms as measured by PANSS (55). This study showed a rapid reduction in negative symptoms with the beneficial effect lasting up to 3 months (55). Further data analysis, published 1 year later, also documented a significant enhancement of insight and beliefs about medication compliance (57).

In terms of the amelioration of negative symptoms, a 36% reduction in the SANS score (54) and a 45% reduction in the PANSS negative score (53) was described in trials using electrode montage with the cathode placed over the right prefrontal cortex. A significant SANS and PANSS reduction were also documented in a study with HD-tDCS stimulation (56).

A study with frontotemporal electrode montage presented a significantly greater reduction in negative symptoms and the total PANSS score after active stimulation compared to the sham (52). As a secondary outcome, the effect of tDCS on cognitive performance was evaluated in the majority of participants, and no beneficial effect was shown in favor of active stimulation over placebo (58).

Detailed information on the studies mentioned in this section is provided in Table 2.

**TABLE 2:** Effects of tDCS on negative symptoms.

Studies	Design	Inclusion criteria, diagnosis	Number of subjects	Electrode placement	Stimulation parameters *(duration, course, total number, intensity used)*	Sham parameters	Outcomes
Gomes et al. 2015 (53)	RDBS, SH	SZ	*n* = 15 (7 active, 8 sham)	Anode – F3 cathode – F4 size: not specified	20 min 1x/day, 2 weeks, weekdays (10) 2 mA	Not specified	PANSS reduction (total score 42.3%, negative score 45.4%, general score 29%) after active tDCS. No effects for CDSS, GAF and PANSSpositive score.
Palm et al. 2016 (54)	RDBS, SH	SZ with predominantly negative symptoms	*n* = 20 (10 active, 10 sham)	Anode – F3 cathode – FP2 size: 7 × 5 cm	20 min 1x/day, 2 weeks, weekdays (10) 2 mA	Not specified	Significantly greater decrease in SANS score (36.1%) and PANSS sum scores (23.4%) after active tDCS compared to sham (0.7, 2.2% respectively). Explorative analysis of fcMRI data revealed changes in subgenual cortex and DLPFC connectivity within fontal-thalamic-temporo-parietal networks. No significant effect of active tDCS on CDSS score or cognitive outcomes.
Chang, et al. 2020 (55)	RDBS, SH	SZ/SZA	*n* = 60 (30 active, 30 sham)	Bilateral: anode 1 – F3/FP1 anode 2 – F4/FP2 reference electrodes – ipsilateral forearm size: 7 × 5 cm	20 min 2x/day, 5 days (10) 2 mA	2 mA for 30 s +110 μA pulse over 15 ms every 550 ms	Rapid reduction of negative symptoms measured by PANSS, with the beneficial effect lasting up to 3 months. Improvement of psychosocial functioning. Improvement of psychopathological symptoms especially for disorganization and cognitive symptoms as measured by the PANSS. No effects on other schizophrenia symptom dimensions or on the performance in neurocognitive tests.
Valiengo et al. 2020 (52)	RDBS, SH	SZ	*n* = 100 (50 active, 50 sham)	Anode – F3 cathode – T3/P3 size: 7 × 5 cm	20 min 2x/day, 5 days (10) 2 mA	2 mA for 30 s + 40 s ramp-up + 40 s ramp-down	Significantly greater improvement in PANSS scores after active tDCS. Higher response rates for negative symptoms in the active group.
Dharani et al. 2021 (56)	RDBS, SH	SZ	*n* = 14 (7 active, 7 sham)	Anode – F3 4 return electrodes (FC1, F7, FC5, AF3) size: 1 cm radius ring electrodes	20 min 2x/day, 5 days (10) 2 mA	1 mA for 30 s	Significant reduction in PANSS, SANS, and CGI-S.


Brunelin et al. 2012 (33)	RDBS, SH	SZ + TR AH	*n* = 30 (15 active, 15 sham)	Anode – F3/FP1 cathode - T3/P3 size: 7 × 5 cm	20 min 2x/day, 5 days (10) 2 mA	2 mA for 40 s +110 μA pulse over 15 ms every 550 ms	Robust AH reduction (mean improvement of 31% in AHRS score) lasting up to 3 months. Amelioration of schizophrenia symptoms as assessed by total PANSS, with significant effect on the negative dimension. Medium effect size on the positive and depressive dimension short of statistical significance.
Fitzgerald et al. 2014 (32)	2x RDBS, SH	SZ/SZA + persistent AH and negative symptoms	*n* = 24 (11 bilateral, 13 unilateral)	Unilateral: anode – F3 cathode – TP3 Bilateral: + anode – F4 + cathode – TP4 size: 35cm^2^	20 min 1x/day, 3 weeks, weekdays (15) 2 mA	2 mA for 30 s with ramp-up	No substantial change in AH, PANSS or SANS score after neither unilateral nor bilateral stimulation.
Smith et al. 2015 (66)	RDBS, SH	SZ/SZA + current smokers	*n* = 37 (19 active, 18 sham)	Anode – F3 cathode – FP2 size: 5,08 cm^2^	20 min 1x/day, 5 days (5) 2 mA	2 mA for 40 s	Significant improvements in MCCB Composite score, and the domain scores for Working Memory and Attention Vigilance with large effect sizes. (MCCB Composite score and domain score for Working Memory remained significant with corrected significance levels). No statistically significant effects on secondary outcome measures (PANSS scores, hallucinations, cigarette craving, or cigarettes smoked).
Fröhlich et al. 2016 (40)	RDBS, SH	SZ/SZA + AH	*n* = 26 (13 active, 13 sham)	Anode – F3/FP1 cathode – T3/P3 (+ return electrode Cz). size: 7 × 5cm	20 min 1x/day, 5 days (5) 2 mA	2 mA for 40 s	A lack of efficacy of active tDCS. A significant reduction in AH not specific to the treatment group. No significant change in PANSS.
Mondino et al. 2016 (35)*^b^*	RDBS, SH	SZ + TR AH	*n* = 23 (11 active, 12 sham)	Anode – F3/FP1 cathode – T3/P3 size: 7 × 5 cm	20 min 2x/day, 5 days (10) 2 mA	2 mA for 30 s	Significant reduction of AH as well as negative symptoms after active tDCS. Reduced resting state functional connectivity (rs-FC) of the left temporoparietal junction with the left anterior insula and the right inferior gyrus, and increased rs-FC of the left TPJ with the left angular gyrus, the left DLPFC and the precuneus after active tDCS.
Shiozawa et al. 2016 (70)	RDBS, SH	SZ	*n* = 10 (5 active, 5 sham)	anode – left DLPFC cathode – right DLPFC size: 35 cm^2^	20 min 2x/day, 5 days 1x “online” tDCS (10) 2 mA + *cognitive training randomly applied during one of the tDCS sessions*	2 mA for 60 s	Failed to demonstrate effect of “online tDCS” on improvement in clinical outcomes (N-back and sequence learning task, PANSS).
Gomes et al. 2018 (69)	RDBS, SH	SZ	*n* = 24 (12 active, 12 sham)	Anode– left DLPFC cathode – right DLPFC size: 25 cm^2^	20 min 1x/day, 2 weeks, weekdays (10) 2 mA	2 mA for 30 s	Without improvement in working memory. Therapeutic effects of tDCS for treatment of persistent symptoms in schizophrenia, with reduction of negative symptoms.
Chang et al. 2018 (37)	RDBS, SH	SZ/SZA + TR AH	*n* = 60 (30 active, 30 sham)	Anode – F3/FP1 cathode – T3/P3 size: 7 × 5 cm	20 min 2x/day, 5 days (10) 2 mA	2 mA for 30 s	No significant changes in the severity of AH or in PANSS after active tDCS. Improvement in the level of insight into illness and into positive symptoms lasting 1 month after active tDCS.
Jeon et al. 2018 (68)	RDBS, SH	SZ	*n* = 56 (28 active, 28 sham)	Anode – F3 cathode – F4 size: 25 cm^2^	30 min 1x/day, 2 weeks, weekdays (10) 2 mA	2 mA for 30 s ramp-up + 30 s ramp-down	MCCB working memory and overall scores improved over time after active tDCS. Depressive symptoms decreased after tDCS. Improvement of PANSS score (did not reach statistical significance).
Koops et al. 2018 (38)	RDBS, SH	TR AH (several diagnostic categories)	*n* = 54 (28 active, 26 sham)	Anode – FP1/F3 cathode – T3/P3 size: 7 × 5 cm	20 min 2x/day, 5 days (10) 2 mA	2 mA for 40 s + 110 μA pulse over 15 ms every 550 ms	Active tDCS was not more effective than placebo on any of the main outcomes (AHRS, PANSS, the Stroop, and the Trail Making Test)
Kantrowitz, J. T. et al. 2019 (39)	RDBS, SH	SZ/SZA + TR AH	*n* = 89 (47 active, 42 sham)	Anode – F3/FP1 cathode – T3/P3 size: 6.75 × 5.75 cm	20 min 2x/day, 5 days (10) 2 mA	2 mA for 40 s ramp-up/ramp-down	Significant reduction in AHRS total score (>30%) across 1-week and 1-month. (Greatest change observed on the AHRS loudness item.) No significant change in PANSS negative.
Lindenmayer et al. 2019 (41)	RDBS, SH	Ultra-TR SZ + persistent AH	*n* = 28 (15 active, 13 sham)	Anode – F3/FP1 cathode – T3/P3 size: 7 × 5 cm	20 min 2x/day, 4 weeks, weekdays (40) 2mA	2 mA for 40 s + 110 μA pulse over 15 ms every 550 ms	Small but meaningful AHRS reduction (21.9%). Significant change in working memory and PANSS total in the active tDCS group. No significant changes in PANSS subscales.
Weickert et al. 2019 (74)	RDBS, SH	SZ/SZA	*n* = 12 (6 active, 6 sham)	Anode – F4 cathode – T3/P3 size: 7 × 5 cm	20 min 1x/day, 4 weeks, weekdays “online” tDCS (20) 2 mA + 2-back test	2 mA for 15 s ramp-up + 15 s ramp-down	Significant improvement in language-based working memory after 2 weeks and verbal fluency after 2 and 4 weeks. No significant effect on any other cognitive assessment. No significant effects on AHRS score.
Smith et al. 2020 (65)	RSBS, SH	SZ + significant cognitive deficit	*n* = 49 (24 active, 25 sham) *45 evaluated	Anode – F3 cathode – FP2 size: 5.08 cm^2^	20 min 1x/day, 2 weeks, weekdays (10) 2 mA	2 mA for 40 s	Significant pro-cognitive effects on some aspects of cognitive testing at 2 and 4 weeks after the final tDCS session (MATRICS Speed of Processing domain). No immediate pro-cognitive effects. No significant effects on other psychiatric outcomes.
Chang et al. 2021 (57)*^a^*	RDBS, SH *ancillary investigation*	SZ/SZA	*n* = 60 (30 active, 30 sham)	Bilateral: anode 1 – F3/FP1 anode 2 – F4/FP2 reference electrodes – ipsilateral forearm size: 7 × 5 cm	20 min 2x/day, 5 days (10) 2 mA	2 mA for 30 s + 110 μA pulse over 15 ms every 550 ms	Significant enhancement of insight levels and beliefs about medication compliance after active tDCS.
Meiron et al. 2021 (71)	RDBS, SH	SZ/SZA	*n* = 19 (11 active, 8 sham, +12 healthy controls for baseline and post-tDCS comparison)	Anode – F3/AF3 cathode – vertex size: 5 × 5 cm	20 min 2x/day, 5 days (10) 2 mA	2 mA for 30 s ramp-up + 30 s ramp-down	Improvement in working memory performance in the active tDCS group. Post-tDCS scores were comparable to healthy control scores. Significant alleviation of symptom severity maintained for 4 weeks.

*Studies placed below the double line did not explore negative symptoms as the primary outcome. AH, auditory hallucinations; AHRS, Auditory Hallucinations Rating Scale; CDSS, Calgary Depression Scale for Schizophrenia; CGI-S, Clinical Global Impression Scale; DLPFC, dorsolateral prefrontal cortex; GAF, Global Assessment of Functioning; MCCB, MATRICS Consensus Cognitive Battery; PANSS, Positive and Negative Syndrome Scale; RDBS, randomized double blind study; RSBS, randomized single blind study; SH, sham controlled; SZ, schizophrenia; SZA, schizoaffective disorder; TPJ, temporoparietal junction; TR, treatment resistant. ^a^Ancillary investigation of secondary outcomes from a previously published study (55).*

### Cognitive Symptoms

Cognitive impairment is one of the main intervention targets in the treatment of schizophrenia. Cognitive impairment evolves even before the onset of schizophrenia (prodromal phase), is observable in most patients in the first episode, often persists during symptomatic remissions, and is relatively stable across time (59). Important domains of cognitive deficit in schizophrenia include deficits in working memory, executive functions, attention, and speech (60). However, generalized impairment of various cognitive functions has been described (61). The search identified twelve RTCs with the primary focus on influencing cognitive functions. The selected studies do not provide entirely consistent results, two-thirds of them, nonetheless, reported at least partial improvement in the observed cognitive domains. Anodal tDCS over the left prefrontal cortex appears to be a promising method for improving cognition in neuropsychiatric disorders (11). Most of the studies chose this type of stimulation with the cathode located above the contralateral orbitofrontal area (62–67), contralateral prefrontal area (68–70), or vertex (71). One study placed the anode over the right prefrontal cortex, and one protocol used bi-anodal and bi-cathodal stimulation in the prefrontal area (72). The current intensity of 2 mA was used in all of the studies, except for one trial which applied a lower current intensity of 1 mA (64).

Three studies investigated the cognitive outcomes after a single application of tDCS (63, 67, 72). Only one of them, using bi-anodal stimulation, reported a positive effect on one of the observed parameters, which was emotion identification (72). A second study remained without any positive effect of tDCS on initially significantly reduced visual processing speed and visual short-term memory storage capacity in patients with schizophrenia, and even considered the possibility that tDCS may interfere with practice-dependent improvements in the rate of visual information uptake (67). A third study described a possible impairment of response inhibition after a single tDCS session (63).

Likewise, two of the protocols with multiple tDCS applications – specifically with bi-frontal electrode placement – did not find a significant effect on cognition (69, 70). However, one of the trials reported therapeutic effects of tDCS for the treatment of persistent symptoms in schizophrenia, with a reduction of negative symptoms (69). The applied electrode positioning was the same as in a similar study from 2015 that was reported previously in the section on negative symptoms (53).

The remaining studies with multiple tDCS applications yielded positive results in affecting cognitive functions. Other positive effects of repeated stimulation regimens included a reduction in the PANSS score (68, 71) and alleviation of depressive symptoms (68). Functional magnetic resonance imaging was acquired during tDCS stimulation from some of the participants in a study with a positive effect on working memory (62). Increased activity in the medial prefrontal cortex below the anode was positively correlated with improved working memory, and decreased activity in the anterior cingulate cortex was associated with improved performance on the executive function task, further suggesting the procognitive effects of tDCS applied over the frontal area (73).

A total of four studies used “online” tDCS application, where stimulation is applied at the time of ongoing cognitive training (62, 64, 70, 74). In two cases, “online” tDCS took place during all (74) or more than half of the cognitive training sessions (64). The remaining studies applied stimulation only during one (70) or two appointments (62). By activating the prefrontal cortex, the trials anticipated augmentation of the cognitive training.

An overview of the tDCS use for cognitive symptoms in schizophrenia is provided in Table 3.

**TABLE 3:** Effects of tDCS on cognitive symptoms.

Studies	Design	Inclusion criteria, diagnosis	Number of subjects (n)	Electrode placement Size of electrodes	Stimulation parameters *(duration, course, total number, intensity used)*	Sham parameters	Outcomes
Rassovsky et al. 2015 (72)	RSBS, SH	SZ	*n* = 36 (12 anodal, 12 cathodal, 12 sham)	Bilateral anodal/cathodal: active electrodes - FP1, FP2 reference electrodes – right arm Size: 7 × 5 cm	20 min anodal/cathodal/sham (1) 2 mA	2 mA for 30 s	Significant improvement in one of the four social cognitive tasks – emotion identification – after anodal stimulation.
Smith et al. 2015 (66)	RDBS, SH	SZ/SZA + current smokers	*n* = 37 (19 active, 18 sham)	Anode – F3 cathode – FP2 size: 5.08 cm^2^	20 min 1x/day, 5 days (5) 2 mA	2 mA for 40 s	Significant improvements in MCCB Composite score and the domain scores for Working Memory and Attention Vigilance with large effect sizes. (MCCB Composite score and domain score for Working Memory remained significant with corrected significance levels). No statistically significant effects on secondary outcome measures (PANSS scores, hallucinations, cigarette craving, or cigarettes smoked).
Nienow et al. 2016 (64)	RSBS, SH	SZ/SZA	*n* = 10	Anode – F3 cathode – right SO size: 7 × 5 cm	20 min 2x/week from 3rd week “online” tDCS (28) 1 mA *Cognitive training 1 h 3x/week for 16 weeks*	Not specified	Suggests “online tDCS” enhances cognitive performance.
Shiozawa et al. 2016 (70)	RDBS, SH	SZ	*n* = 10 (5 active, 5 sham)	Anode – left DLPFC cathode – right DLPFC size: 35 cm^2^	20 min 2x/day, 5 days 1x “online” tDCS (10) 2 mA + *cognitive training randomly applied during one of the tDCS sessions*	2 mA for 60 s	Failed to demonstrate effect of “online tDCS” on improvement in clinical outcomes (N-back and sequence learning task, PANSS).
Gögler et al. 2017 (67)	RDBS, SH	SZ/SZA	*n* = 20 patients (10 active, 10 sham) *n* = 20 healthy controls (10 active, 10 sham)	Anode – F3 cathode –FP2 size: 35 cm^2^	20 min (1) 2 mA	2 mA for 30 s + 15 s ramp-up + 15 s ramp-down	Prefrontal tDCS may interfere with practice-dependent improvements in the rate of visual information uptake.
Orlov et al. 2017 (62)	RDBS, SH	SZ/SZA	*n* = 49 (24 active, 25 sham)	Anode – F3 cathode – FP2 size: 35 cm^2^	30 min on days 1 and 14 “online” tDCS (2) 2 mA *Cognitive training 2x/day on days 1, 2, 14, 56*	2 mA for 30 s	Significant long-term effect of tDCS on working memory (suggested effect on consolidation of learning, no significant benefit during the acute stimulation on working memory).
Gomes et al. 2018 (69)	RDBS, SH	SZ	*n* = 24 (12 active, 12 sham)	Anode– left DLPFC cathode – right DLPFC size: 25 cm^2^	20 min 1x/day, 2 weeks, weekdays (10) 2 mA	2 mA for 30 s	Without improvement in working memory. Therapeutic effects of tDCS for treatment of persistent symptoms in schizophrenia, with reduction of negative symptoms.
Jeon et al. 2018 (68)	RDBS, SH	SZ	n = 56 (28 active, 28 sham)	anode – F3 cathode – F4 size: 25 cm^2^	30 min 1x/day, 2 weeks, weekdays (10) 2 mA	2 mA for 30 s ramp-up + 30 s ramp-down	MCCB working memory and overall scores improved over time after active tDCS. Depressive symptoms decreased after tDCS. Improvement of PANSS score (did not reach statistical significance).
Weickert et al. 2019 (74)	RDBS, SH	SZ/SZA	*n* = 12 (6 active, 6 sham)	anode – F4 cathode – T3/P3 size: 7 × 5 cm	20 min 1x/day, 4 weeks, weekdays “online” tDCS (20) 2 mA + 2-back test	2 mA for 15 s ramp-up + 15 s ramp-down	Significant improvement in language-based working memory after 2 weeks and verbal fluency after 2 and 4 weeks. No significant effect on any other cognitive assessment. No significant effects on AHRS score.
Smith et al. 2020 (65)	RSBS, SH	SZ + significant cognitive deficit	*n* = 49 (24 active, 25 sham) *45 evaluated	Anode – F3 cathode – FP2 size: 5.08 cm^2^	20 min 1x/day, 2 weeks, weekdays (10) 2 mA	2 mA for 40 s	Significant pro-cognitive effects on some aspects of cognitive testing at 2 and 4 weeks after the final tDCS session (MATRICS Speed of Processing domain). No immediate pro-cognitive effects. No significant effects on other psychiatric outcomes.
Meiron et al. 2021 (71)	RDBS, SH	SZ/SZA	*n* = 19 (11 active, 8 sham, +12 healthy controls for baseline and post-tDCS comparison)	Anode – F3/AF3 cathode – vertex size: 5 × 5 cm	20 min 2x/day, 5 days (10) 2 mA	2 mA for 30 s ramp-up + 30 s ramp-down	Improvement in working memory performance in the active tDCS group. Post-tDCS scores were comparable to healthy control scores. Significant alleviation of symptom severity maintained for 4 weeks.
Schilling et al. 2021 (63)	RDBS, SH	SZ/SZA/ATPD	*n* = 48 (24 active, 24 sham)	Anode – F3 cathode – FP2 size: 5 × 5 cm	20 min (1) 2 mA	2 mA for 40 s	No acute enhancement of executive functions. Impaired performance in the response inhibition task within 20 min after the stimulation.


Palm et al. 2016 (54)	RDBS, SH	SZ with predominantly negative symptoms	*n* = 20 (10 active, 10 sham)	Anode – F3 cathode – FP2 size: 7 × 5 cm	20 min 1x/day, 2 weeks, weekdays (10) 2 mA	Not specified	Significantly greater decrease in SANS score (36.1%) and PANSS sum scores (23.4%) after active tDCS compared to sham (0.7, 2.2% respectively). Explorative analysis of fcMRI data revealed changes in subgenual cortex and DLPFC connectivity within fontal-thalamic-temporo-parietal networks. No significant effect of active tDCS on CDSS score or cognitive outcomes.
Orlov et al. 2017 (73)*^a^*	RDBS, SH *fMRI study as a part of a larger behavioral study*	SZ/SZA	n = 49 (24 active, 25 sham)	anode – F3 cathode – FP2 size: 35 cm^2^	30 min on days 1 and 14 “online” tDCS (2) 2 mA *Cognitive training 2x/day on days 1, 2, 14, 56*	2 mA for 30 s	Modulation of functional activation in local task-related regions and in more distal nodes in the network with active tDCS.
Chang, et al. 2018 (37)	RDBS, SH	SZ/SZA + TR AH	*n* = 60 (30 active, 30 sham)	Anode – F3/FP1 cathode – T3/P3 size: 7 × 5 cm	20 min 2x/day, 5 days (10) 2 mA	2 mA for 30 s	No significant changes in the severity of AH or in PANSS after active tDCS. Improvement in the level of insight into illness and into positive symptoms lasting 1 month after active tDCS.
Koops et al. 2018 (38)	RDBS, SH	TR AH (several diagnostic categories)	*n* = 54 (28 active, 26 sham)	anode - FP1/F3 cathode - T3/P3 size:7 × 5 cm	20 min 2x/day, 5 days (10) 2 mA	2 mA for 40 s + 110 μA pulse over 15 ms every 550 ms	Active tDCS was not more effective than placebo on any of the main outcomes (AHRS, PANSS, the Stroop, and the Trail Making Test)
Chang et al. 2019 (42)*^b^*	RDBS, SH *ancillary analysis*	SZ/SZA + TR AH	*n* = 60 (30 active, 30 sham)	Anode – F3/FP1 cathode – T3/P3 size: 7 × 5 cm	20 min 2x/day, 5 days (10) 2 mA	2 mA for 30 s	Significant trends in PANSS total and general scores after active tDCS, does not reach statistical significance compared to sham stimulation. No significant effects on other psychopathological symptoms and psychosocial functioning. A trend-level improvement of planning ability.
Lindenmayer et al. 2019 (41)	RDBS, SH	Ultra-TR SZ + persistent AH	*n* = 28 (15 active, 13 sham)	Anode – F3/FP1 cathode – T3/P3 size: 7 × 5 cm	20 min 2x/day, 4 weeks, weekdays (40) 2mA	2 mA for 40 s + 110 μA pulse over 15 ms every 550 ms	Small but meaningful AHRS reduction (21.9%). Significant change in working memory and PANSS total in the active tDCS group. No significant changes in PANSS subscales.
Chang et al. 2020 (55)	RDBS, SH	SZ/SZA	*n* = 60 (30 active, 30 sham)	Bilateral: anode 1 – F3/FP1 anode 2 – F4/FP2 reference electrodes – ipsilateral forearm size: 7 × 5 cm	20 min 2x/day, 5 days (10) 2 mA	2 mA for 30 s + 110 μA pulse over 15 ms every 550 ms	Rapid reduction of negative symptoms measured by PANSS, with the beneficial effect lasting up to 3 months. Improvement of psychosocial functioning. Improvement of psychopathological symptoms especially for disorganization and cognitive symptoms as measured by the PANSS. No effects on other schizophrenia symptom dimensions or on the performance in neurocognitive tests.
Kao et al. 2020 (43)*^c^*	RDBS, SH *ancillary analysis*	SZ/SZA + TR AH	*n* = 60 (30 active, 30 sham)	Anode – F3/FP1 cathode – T3/P3 size: 7 × 5 cm	20 min 2x/day, 5 days (10) 2 mA	2 mA for 30 s + 110 μA pulse over 15 ms every 550 ms	Brief optimization of self-reported insight levels, beliefs about treatment adherence, and psychological domain of life quality after active tDCS.
Bulubas et al. 2021 (58)*^d^*	RDBS, SH *ancillary analysis*	SZ	*n* = 100 (50 active, 50 sham) *90 patients included in ancillary analysis* *(48 active, 42 sham)*	anode – F3 cathode – T3/P3 size: 7 × 5 cm	20 min 2x/day, 5 days (10) 2 mA	2 mA for 30 s + 40 s ramp-up + 40 s ramp-down	No beneficial effects of active tDCS over sham in any of the cognitive tests. Improvements of executive functions and delayed memory in favor of sham stimulation.

*Studies placed below the double line did not explore cognitive symptoms as the primary outcome. AH, auditory hallucinations; AHRS, Auditory Hallucinations Rating Scale; ATPD, acute transient psychotic disorder; DLPFC, dorsolateral prefrontal cortex; fMRI, functional magnetic resonance imaging; MCCB, MATRICS Consensus Cognitive Battery; PANSS, Positive and Negative Syndrome Scale; RDBS, randomized double blind study; RSBS, randomized single blind study; SH, sham controlled; SO, supraorbital; SZ, schizophrenia; SZA, schizoaffective disorder; TR, treatment resistant. ^a^Part of a larger behavioral study (73). ^b^Ancillary analysis of secondary outcomes from a previously published study (42). ^c^Ancillary analysis of secondary outcomes from a previously published study (43). ^d^Ancillary data analysis of part of the subjects from a previously published study (58).*

### Transcranial Direct Current Stimulation Tolerability and Side Effects

None of the studies reported any serious adverse effects. The most common side effects (SE) documented in the trials included skin redness, tingling or itching sensation under the electrodes, moderate fatigue, tiredness, and headache, all of which were usually well-tolerated and of a mild and transient character. Mostly, there was no significant difference in frequency of SE between active and sham tDCS groups, except for skin redness and a burning sensation under the electrodes with higher frequency in the active tDCS group, which was documented in some of the papers (38, 52, 65). According to recent reviews, there is no evidence for irreversible injury produced by conventional tDCS protocols within a wide range of stimulation parameters (75) and within standard protocols, tDCS is considered a safe method (76).

### Risk of Bias

The Cochrane risk of bias assessment tool was used to evaluate the methodological quality of each trial. Study quality assessment included randomization process, deviations from the intended interventions, missing outcome data, measurement of the outcome, and selection of the reported result. Each domain was scored as “low risk,” “some concerns,” or “high risk.” The level of the risk of bias varied across studies. The most common potential causes of bias were the insufficient description of the randomization and blinding process, dealing with missing data, and unavailability of pre-specified analysis plan (i.e., study protocol) which led to scoring as “some concerns.” A significant number of studies did not provide a sufficient description of blinding of staff delivering intervention and assessors of outcome measures, or did not describe the method used for randomization other than stating the participants were randomized (33–35, 37, 41, 53, 62, 64, 70–72). One of the studies reported only partial effectiveness of blinding since both subjects and testers could correctly guess that the sham group received sham stimulation in most of their guesses (65). There was also missing outcome data in a larger part of the trials mainly due to discontinuation of participants, leading to scoring as “some concerns” in the domain (37, 38, 41, 53, 56, 62, 64–66, 68–72, 74). None of the reviewed studies scored “high” in the overall risk of bias.

## Discussion

This is an up-to-date review article offering a cross-section of current research with a focus on tDCS application in schizophrenia. Following a standardized literature search, we identified 27 randomized controlled trials. A total of 966 patients diagnosed with schizophrenia or schizoaffective disorder participated in these clinical trials. As a primary aim, ten trials examined the effects of tDCS on positive symptoms with six of them yielding positive results. All five trials focusing primarily on negative symptoms showed some improvement in the measured outcomes. Twelve trials explored the impact of tDCS on cognitive functions and out of those, eight trials report beneficial effects in at least one measured aspect of cognition. Overall, we could not establish a reporting bias.

The reviewed studies differed considerably in the experimental design and stimulation protocols. However, all of the clinical trials selected the anode placement to be over the prefrontal cortex. This may be explained by the effort to positively influence the aforementioned attenuated prefrontal activity that is seen in patients with schizophrenia. Nonetheless, the differences between the cortical activity in early-course and chronic schizophrenia may be of consideration in connection to the anode placement. A previous study suggests a difference in the activity of the prefrontal cortex among early-course schizophrenic patients in contrast to attenuation of the activity that is commonly described in patients with chronic illness (77). In such cases, the activation of prefrontal regions by the anodal transcranial stimulation may not be beneficial. None of the reviewed studies reported using neuronavigation for exact electrode positioning, most of them referred to the 10-20 EEG system frequently used for tDCS electrode montage. Nonetheless, due to inter-personal brain variability this method leads to limited targeting accuracy (78). Although neuronavigation methods are not commonplace outside highly specialized research centers, their future implementation could mean achieving more effective stimulation and consequently better clinical outcomes. Another element of tDCS application to consider is the duration of active stimulation. The vast majority of the selected trials used 20 min of stimulation, and only three of them chose to prolong the stimulation up to 30 min. Current research suggests that the effect of anodal tDCS may not be directly proportional to the duration of the active stimulation, and may on the contrary decrease or even reverse with prolonged stimulation (79, 80). However, the studies included in the review that used a 30-min protocol provided positive outcomes with all of them focusing on cognitive measures. Further exploration of the exact electrode placement and duration of the stimulation should be considered in future studies. Additionally, number of sessions in repeated tDCS application protocols and the repetition interval is an important factor to examine. Studies included in this review used stimulation protocols with tDCS applied once or twice daily, usually separating the two stimulation sessions by 2–3 h. Twice-a-day stimulation is used in order to strengthen the effect. Repeated application protocols offer significant opportunities for induction of long-lasting and significant neuroplastic change (81). However, specific timing of repetition intervals is important for optimizing cumulative effects of tDCS (82). Previously published studies indicate that short repetition interval (<30 min) can lead to prolongation of tDCS after-effects (81–83), whereas longer repetition interval (3 or 24 h) result in no excitability-enhancing after effects or can nullify them (83, 84). Current research focuses on accelerated tDCS protocols (85, 86), and future RCT protocols using tDCS as a treatment option for schizophrenia might benefit from their implementation.

In recent years, the emphasis is also placed on gender differences. Brain anatomy, chemistry, and function differ in relation to sex, leading to differences in response to neurostimulation methods in men and women (87, 88). These issues have been addressed and explored in recent studies (89–91). Some of the studies included in this review controlled for potential confounding effects of male to female ratio in trial groups, however, none of them specifically focused on the various effects of tDCS in connection to gender. There was also no consideration of altering stimulation protocols according to sex-related brain differences or examining response to tDCS in women and men separately. In the future, closer exploration of gender-tailored stimulation protocols might be of interest.

The clinical trials included in the review considerably differ in sample size. As the outcomes are not consistent, this makes it difficult to offer clear recommendations for future research. The differences in methodologies, experimental design, and protocols are considerable limitations for selecting an appropriate and most effective design for future trials. More studies with a clear design and robust sample size are needed to better evaluate the clinical effects and possible application of tDCS in treating patients with schizophrenia.

This review has several limitations. Firstly, only randomized controlled double-blind parallel trials were included, which decreased the total number of reviewed studies. Open-label and cross-over studies may play an important role in an overall assessment of tDCS efficacy, and future reviews may consider their inclusion. Secondly, we did not perform a meta-analysis of the selected research, as this article only brings a qualitative overview, and therefore statistical data are not offered for the overall assessment.

## Conclusion

This review provides a summary of current research on tDCS application in patients suffering from schizophrenia. Albeit the 2017 guidelines (16) exclude some of the sources on the use of tDCS in schizophrenia as poor evidence, current guidelines list tDCS as a Level B (Probably effective) therapeutic method for the treatment of AH and positive/negative symptoms (24). This review also focused on tDCS application as a treatment for cognitive schizophrenic symptoms, where tDCS appears to be a promising therapeutic method. However, ongoing research is needed to confirm these conclusions and to further specify distinct application parameters.

## Author Contributions

ZS: conceptualization, methodology, investigation, and writing—original draft preparation. MK: conceptualization, methodology, investigation, supervision, writing—reviewing, and editing. Both authors: contributed to the article and approved the submitted version.

## Conflict of Interest

ZS was employed by the company Hospital České Budĕjovice, a.s. The remaining author declares that the research was conducted in the absence of any commercial or financial relationships that could be construed as a potential conflict of interest.

## Publisher’s Note

All claims expressed in this article are solely those of the authors and do not necessarily represent those of their affiliated organizations, or those of the publisher, the editors and the reviewers. Any product that may be evaluated in this article, or claim that may be made by its manufacturer, is not guaranteed or endorsed by the publisher.

## Supplementary Material

Supplementary Figure 1 PRISMA 2020 flow diagram for new systematic reviews, which included searches of databases, registers, and other sources [A LiteratureMini-Review of Transcranial Direct Current Stimulation (tDCS) in Schizophrenia]. From: Page MJ, McKenzie JE, Bossuyt PM, Boutron I, Hoffmann TC, Mulrow CD, et al. The PRISMA 2020 statement: an updated guideline for reporting systematic reviews. *BMJ* 2021;372:n71. doi: 10.1136/bmj.n71. For more information, visit http://www.prisma-statement.org/.
