# Supplementary figures and images for: A Literature Mini-Review of Transcranial Direct Current Stimulation in Schizophrenia
Source: Front Psychiatry. 2022 Apr 21;13:874128. doi: 10.3389/fpsyt.2022.874128 (PMC9069055; doi:10.3389/fpsyt.2022.874128)

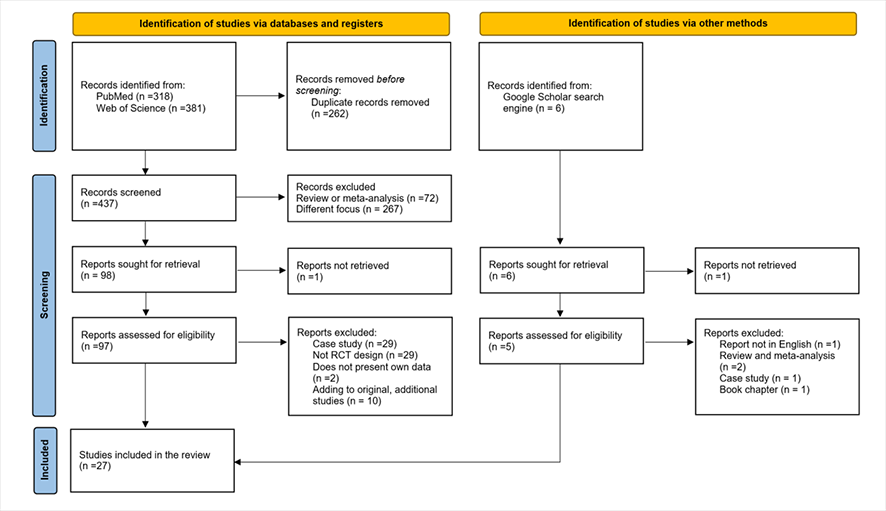

Supplement: Supplementary Figure 1 — PRISMA 2020 flow diagram for new systematic reviews, which included searches of databases, registers, and other sources [A LiteratureMini-Review of Transcranial Direct Current Stimulation (tDCS) in Schizophrenia]. From: Page MJ, McKenzie JE, Bossuyt PM, Boutron I, Hoffmann TC, Mulrow CD, et al. The PRISMA 2020 statement: an updated guideline for reporting systematic reviews. BMJ 2021;372:n71. doi: 10.1136/bmj.n71. For more information, visit http://www.prisma-statement.org/. [file Image_1.TIF]
